# Caco-2 Cell Acquisition of Dietary Iron(III) Invokes a Nanoparticulate Endocytic Pathway

**DOI:** 10.1371/journal.pone.0081250

**Published:** 2013-11-21

**Authors:** Dora I. A. Pereira, Bianca I. Mergler, Nuno Faria, Sylvaine F. A. Bruggraber, Mohamad F. Aslam, Lynsey K. Poots, Laura Prassmayer, Bo Lönnerdal, Andy P. Brown, Jonathan J. Powell

**Affiliations:** 1 Medical Research Council Human Nutrition Research (MRC HNR), Elsie Widdowson Laboratory, Cambridge, United Kingdom; 2 Department of Nutrition, University of California Davis, Davis, California, United States of America; 3 Institute for Materials Research, School of Process, Environmental and Materials Engineering, University of Leeds, Leeds, United Kingdom; University of Birmingham, United Kingdom

## Abstract

Dietary non-heme iron contains ferrous [Fe(II)] and ferric [Fe(III)] iron fractions and the latter should hydrolyze, forming Fe(III) oxo-hydroxide particles, on passing from the acidic stomach to less acidic duodenum. Using conditions to mimic the *in vivo* hydrolytic environment we confirmed the formation of nanodisperse fine ferrihydrite-like particles. Synthetic analogues of these (~ 10 nm hydrodynamic diameter) were readily adherent to the cell membrane of differentiated Caco-2 cells and internalization was visualized using transmission electron microscopy. Moreover, Caco-2 exposure to these nanoparticles led to ferritin formation (i.e., iron utilization) by the cells, which, unlike for soluble forms of iron, was reduced (*p*=0.02) by inhibition of clathrin-mediated endocytosis. Simulated lysosomal digestion indicated that the nanoparticles are readily dissolved under mildly acidic conditions with the lysosomal ligand, citrate. This was confirmed in cell culture as monensin inhibited Caco-2 utilization of iron from this source in a dose dependent fashion (*p*<0.05) whilet soluble iron was again unaffected. Our findings reveal the possibility of an endocytic pathway for acquisition of dietary Fe(III) by the small intestinal epithelium, which would complement the established DMT-1 pathway for soluble Fe(II).

## Introduction

Dietary non-heme iron (Fe) contains ferrous [Fe(II)] and ferric [Fe(III)] fractions in a number of chemical forms: for example Fe in ferritin and in Fe-sulphur cluster proteins [1–3]. Redox changes to dietary Fe between the points of ingestion and cellular uptake from the intestinal lumen are complex and may favour oxidation to Fe(III) due to intestinal pH and endogenous ligands, or reduction to Fe(II) due to the presence of ascorbate and low luminal oxygen levels [4,5]. Overall, however, both Fe(II) and Fe(III) appear to reach the intestinal epithelial surface to some extent [4,6–8]. Fe(II) is transported by the divalent metal transporter-1 (DMT-1) and Fe(III) is proposed to undergo enzymatic reduction to Fe(II), perhaps by duodenal cytochrome B (DcytB), prior to transport by DMT-1 [9–13]. Chemically, however, there is an uncomfortable paradox with respect to this latter pathway. Fe(III) is remarkably insoluble at duodenal pH, immediately precipitating out of solution as Fe(III) poly oxo-hydroxide:

 [logK_eq_ Fe^3+^  +  3  H_2_O  ↔  Fe(OH)_3_  +  3H^+^ =  -11.7 [14]].

To obviate iron precipitation several authors have suggested the presence of endogenous Fe binding agents (i.e. proteins, organic acids etc.) in gastrointestinal secretions that retain Fe(III) in a soluble form. The aluminium ion (Al(III)) can be viewed as a kinetically less labile, non-paramagnetic metal ion (M) ‘probe’ for Fe(III). Work on the chemical speciation of Al(III) in the intestinal lumen did not identify any endogenous ligands that could prevent its precipitation as an oxo-hydroxide at peri-neutral pH [15]. Instead, Powell et al. proposed that a range of endogenous, low molecular weight ligands could slow the rate of hydrolysis to allow M(III) binding to gastrointestinal mucins [15]. Indeed, luminal Fe binding to gastrointestinal mucins has been identified by a number of authors [8,16,17] and so effective is this binding that gastric mucin has been previously referred to as ‘gastroferrin’: the endogenous gastro(intestinal) molecule that can keep dietary Fe(III) in ‘solution’ [18–24]. Rudzki et al. detailed the nature of the binding between Fe(III) and the Fe-binding glycoprotein of gastric juice (mucin) and showed that at pH values of the proximal bowel (typical pH range 5.8-6.7 [25]), Fe(III) is not quite kept in ‘solution’ [26].Instead, it appears that Fe(III) commences hydrolysis but further growth and agglomeration of the Fe(III) poly oxo-hydroxide nanoparticles are prevented by mucin binding [26]. They visualized the nanoparticles formed in the Fe-glycoprotein complex, noting that they were ~10-20 nm in diameter and appeared to be amorphous aggregates. The Fe(III) oxo-hydroxide phase expected to first precipitate from an Fe(III)-bearing solution at proximal small bowel pH is ferrihydrite [27]. This is a cross-linked polymeric (i.e. poorly (nano)crystalline) phase with primary platelets of 2-5 nm in size depending on the degree of crystallinity and is prone to agglomeration and aggregation [28]. Rudzki et al. produced a synthetic version, referring to this phase as colloidal Fe and, importantly, showed that direct instillation of this ferrihydrite–mucin solution into the duodenum of rats, led to Fe absorption nearly equivalent to that of the gold standard, Fe(II) ascorbate [26]. 

How nanoparticulate Fe(III) poly oxo-hydroxide, which is insoluble at duodenal pH, would be acquired by enterocytes and processed for absorption and utilization is not clear. Current understanding of dietary Fe(III) absorption suggests synergistic association between an intestinal ferrireductase (e.g. DcytB) and the apical Fe(II) transporter ( DMT-1) such that the enzyme reduces Fe(III) allowing its transport and absorption. However, our current *in vivo* studies reasonably preclude the requirement for reduction and dissolution of Fe(III) poly oxo-hydroxide prior to uptake by the enterocyte (Pereira, Latunde-Dada and Powell; unpublished observations). To investigate the above paradox in more detail we first built on the model of Rudzki et al. [26,29] using mucin plus typical low molecular weight ligands of the gastrointestinal lumen to better mimic *in vivo* Fe(III) hydrolysis. We confirmed the formation of a fine ferrihydrite-like phase in ‘luminally hydrolysed’ dietary Fe(III) and then we probed cellular uptake and utilization of synthetic ligand-modified ferrihydrite, as an analogue for this nanoparticulate phase, demonstrating the requirement of endocytic uptake mechanisms. 

## Materials and Methods

### Synthesis of iron materials

Soluble Fe(II) material was prepared by mixing an acidified stock solution of Fe(II) sulphate heptahydrate (40 mM) with a stock solution of ascorbic acid (0.5 M) to achieve a molar ratio of 1:100 (Fe:ascorbic acid). Soluble Fe(III) maltol chelate (Fe(III) maltol) was produced by mixing a stock solution of Fe(III) chloride (8 mM) with a maltol (3-hydroxy-2-methyl-4H-pyran-4-one) solution (40 mM) to achieve a molar ratio of Fe:maltol of 1:5. Soluble Fe(III) nitrilotriacetate chelate (Fe(III) NTA) was produced by mixing a solution of Fe(III) chloride (8 mM) with a NTA solution to achieve a molar ratio of Fe:NTA of 1:5. The pH of the above mixtures was adjusted to 7.4 with NaOH prior to use. Ligand-modified (LM) Fe(III) poly oxo-hydroxide material was produced following the protocol described by Powell et al. [30]. Briefly, an acidic concentrated stock solution of Fe(III) chloride (40 mM) was added to a solution containing tartaric acid and adipic acid or, in the case of un-modified Fe(III) oxo-hydroxide, to 0.9 %(w/v) of electrolyte (potassium chloride). The initial pH of the mixture was always below 2.0 and the iron was fully solubilized. The pH was then slowly increased by drop-wise addition of a concentrated solution of NaOH with constant agitation until the desired final pH (ca. 7.4 for LM Fe(III) poly oxo-hydroxide and 7.4-8.2 for un-modified Fe(III) oxo-hydroxide) were attained. In the case of LM Fe(III) poly oxo-hydroxide the ratio of Fe:tartaric acid:adipic acid in the final suspension was 2:1:1. 

### Chemical characterisation

Detailed methods of the below are provided in the Supplementary Methods S1. Fe(III) structures were characterised by transmission electron microscopy (TEM) after hydrolysis of Fe(III) in simulated digestion medium. The solubility of LM Fe(III) poly oxo-hydroxide and un-modified Fe(III) poly oxo-hydroxide (i.e. standard synthetic ferrihydrite) was determined at pH 5.0 ± 0.1 in a 10 mM citric acid, 0.15 M NaCl solution. The Fe material was added to the assay solution at an Fe concentration of ca. 1 mM and incubated for 360 min at room temperature. Soluble iron was determined following ultrafiltration (3,000 Da MWCO). The hydrodynamic particle size of the nanoparticulate LM Fe(III) poly oxo-hydroxide material was determined by Dynamic Light Scattering (DLS) and the non-aquated primary particle size by Transmission Electron Microscopy (TEM). 

### Cellular uptake studies

To avoid aggregation/agglomeration of the nanoparticulate iron, the medium for cellular uptake consisted of a balanced salt solution (BSS) containing 130 mM NaCl, 10 mM KCl, 1 mM MgSO_4_, 5 mM Glucose and 1 mM CaCl_2_ in 10 mM PIPES buffer (pH 7.4). Immediately before the cellular uptake experiments, fresh solutions of the Fe materials were prepared in BSS at an Fe concentration of 200 μM and the partition of the Fe into the soluble, nanoparticulate and microparticulate fractions was assessed to assure that most of the Fe (i.e. >90%) was present in the nanoparticulate fraction and had not agglomerated/aggregated. DLS measurements were also taken of the nanoparticulate fraction to assure a mono-disperse distribution of the intended size (i.e. ~10nm). Detailed methodology is shown in Methods S1.

### Iron uptake in undifferentiated Caco-2 cells

Human adenocarcinoma (Caco-2) cells were obtained from ATCC (LGC standards, Middlesex, United Kingdom). Cells were seeded at 1.13 x 10^6^ cells/mL onto 6-well cell culture plates. Plates were centrifuged at 680 *xg* for 5 min to remove the growth medium. The different Fe preparations in uptake medium were carefully added to the cells and incubated for 1 h at 37° C. Uptake medium with no supplemented Fe was also incubated with cells as a control. Each condition was tested in triplicate wells for each experiment. After the Fe incubation period, the uptake medium was removed by centrifugation and aspiration, and the cells were washed with phosphate buffered saline (PBS)-EDTA (2 mM) to remove any loosely bound Fe. The cells were then resuspended in fresh MEM (with no supplemental Fe) and returned to the incubator for an additional 23 h to allow for ferritin-protein formation to plateau [31]. At the end of the incubation period, the cells were washed with Dulbecco's Phosphate-Buffered Saline (DPBS; PAA Laboratories) and lysed with Mammalian Protein Extraction Reagent (MPER^®^, Thermo Fisher Scientific, Cramlington, UK). After lysis, cell debris was removed by centrifugation (5 min, 16,000 *xg*) and the supernatant used for analysis. 

### Iron uptake in differentiated Caco-2 cells

Cells (Caco-2 ATCC as above) were seeded at ca. 1.8 x 10^5^ cells/mL onto 6-well cell culture plates. The Caco-2 cells were maintained under the culture conditions described in Methods S1 and used for the Fe uptake experiments at 11 or 12 days post-seeding. Cell differentiation and formation of a cell monolayer were confirmed for this timepoint as described in Methods S1. Approximately 16 h prior to the experiments the growth medium was replaced by non-supplemented MEM, i.e. without FBS or antibiotics, and cells were returned to the incubator. This was carried out to ‘starve’ cells of Fe prior to the experiments and did not induce any changes in the cell monolayer integrity as confirmed by TEER measurements. The Caco-2 cell monolayer was then washed once with pre-warmed DPBS and incubated with the different Fe materials or control uptake medium for 1 h at 37° C. For each experiment every condition was investigated in triplicate wells. Following the Fe incubation period the uptake medium was removed, the cell monolayer was washed three times with PBS-EDTA (2 mM) to remove any Fe loosely adherent to the cell membrane and fresh non-supplemented MEM was added. The cells were then returned to the incubator for an additional 23 h to allow for ferritin-protein formation [31]. At the end of the incubation period, the cells were washed and lysed as before. The cell lysate supernatant was used for analysis. 

The Caco-2 cells requirement of energy for Fe uptake from Fe(III) maltol or the nanoparticulate LM Fe(III) poly oxo-hydroxide was investigated by low temperature incubation. The cells were kept on ice for 10 min prior to the experiment and during the 1 h incubation period with the two Fe materials suspended in uptake medium. The cells were then washed 3 times with ice-cold PBS-EDTA (2 mM) and fresh non-supplemented MEM was added and the cells incubated for the remaining 23 h at 37° C and treated as above.

To study the effect of endocytosis-related-pathway-inhibitors on Fe uptake from the different materials, Caco-2 cells were co-incubated for 1 h at 37° C with the Fe materials in BSS supplemented with the following inhibitors (each inhibitor tested in triplicate wells): chlorpromazine (100 µM), filipin (5 mg/L), methyl-β-cyclodextrin (5 mM). Additionally, cells were incubated with Fe in K^+^-free BSS consisting of 130 mM NaCl, 1 mM MgSO_4_, 5 mM glucose and 1 mM CaCl_2_ in 10 mM PIPES buffer (pH 7.4). For this experimental condition cells were pre-incubated for 5 min with a 1:1 mixture of Dulbecco's Modified Eagle Medium (DMEM):water prior to incubation in K^+^-free medium. For the cellular lysosomal dissolution experiments, cells were co-incubated for 1 h at 37° C with the Fe materials in BSS supplemented with monensin (5-30 µM). As before ferritin-formation was assessed at 23h following the removal of the inhibitors and Fe materials. Controls were performed for each inhibitor by incubating the cells with uptake medium (BSS) supplemented with the inhibitor but not containing Fe.

Chlorpromazine [32,33] and K^+^ free BSS [33,34] were used to inhibit clathrin-mediated uptake. Filipin [35] and methyl-β-cyclodextrin [34,36] were used to inhibit caveolin-mediated uptake. Monensin was used to inhibit acidification of endosomes/lysosomes [37–39].

### Analysis

Cell lysate supernatants were analysed for cellular ferritin-protein content using the commercial enzyme-linked immunosorbent assay kit “Spectro Ferritin” (ATI Atlas, Chichester, UK). The uptake data were normalized to total cell protein content determined with the non-interfering protein assay^™^ (NIPA^™^, Calbiochem/Merck, Nottingham, UK). TEER measurements were carried out to determine if the chemical inhibitors used in the uptake studies had a negative impact on the integrity of the cell monolayer (an early indication of toxicity) as detailed in Methods S1. Electron microscopy was used to visualize nanoparticulate Fe uptake by the Caco-2 cells as detailed in Methods S1. 

### Statistical analysis

All statistical analysis was performed using GraphPad Prism version 6 for Windows (GraphPad Software, San Diego, California, USA). Unless stated otherwise, results are presented as means with standard deviations (s.d.). The unpaired t-test was used to statistically compare ferritin-protein formation for the different Fe compounds in differentiated and undifferentiated cells and ferritin-protein formation with and without chemical inhibitors of endocytosis-related pathways (level of significance set to *p*<0.05). The repeated measures 2-way ANOVA with Sidak correction for multiple comparisons was used to statistically compare TEER at different time points. Regular 2-way ANOVA (no matching) was used to statistically compare ferritin formation for different monensin doses between the nano Fe and the soluble Fe materials. Ferritin-protein concentration in the presence of monensin was fitted with a non-linear dose-response inhibition curve (i.e. Log_10_(inhibitor) versus response).

## Results and Discussion

### Formation and cellular uptake of ligand-modified Fe(III) poly oxo-hydroxide nanoparticles

**Figure 1:**
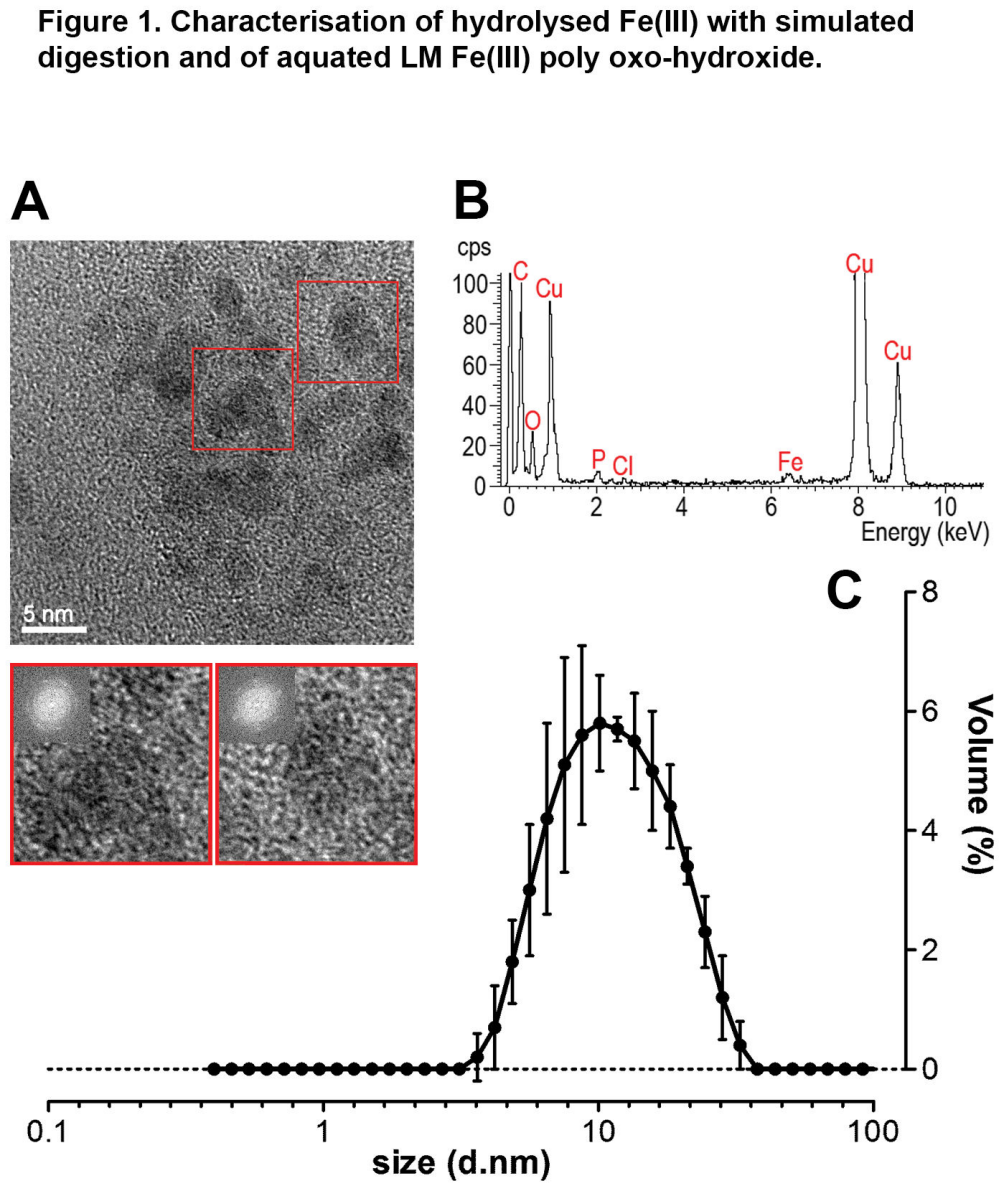
Characterisation of hydrolysed Fe(III) with simulated digestion and of aquated LM Fe(III) poly oxo-hydroxide. **A**, Transmission Electron Microscopy (TEM) images collected from a drop of suspension after simulated digestion of 1 mM Fe(III) chloride in the presence of 2 g/L mucin and low molecular weight ligands. The boxed regions are shown magnified below and highlight the presence of fine, poorly crystalline nanoparticles dispersed in an amorphous gel. Crystallinity is indicated by the spots in the inset diffractograms (fast Fourier transforms) in the boxed regions and lattice spacings are discussed in the main text. Scale bar represents 5 nm. **B**, Whole area EDX analysis of a particle agglomerate similar to those in ‘A’ shows elemental compositions (the specimen support film and grid produce the background C and Cu signals respectively). **C**, Hydrodynamic size distribution of nanoparticulate 500 µM LM Fe(III) poly oxo-hydroxide in balanced salt solution (BSS) measured by Dynamic Light Scattering (DLS). Values are expressed as mean diameter ± s.d. (3 independent measurements) on a log_10_ scale.

First, as a mimetic of gastrointestinal hydrolysis conditions, Fe(III) chloride was dissolved at pH 1.2 in a solution of relevant, low molecular weight ligands and gastric porcine mucin and then adjusted to pH 6.5-7.1 using sodium bicarbonate (final Fe concentration 1 mM) (detailed methodology is available in Methods S1). A drop of the resultant ‘solution’ (colloidal suspension) was placed on a thin amorphous carbon film for TEM and imaged. Agglomerates of fine (~5 nm) poorly crystalline particles in an amorphous gel were noted (Figure 1A). Whole area energy dispersive X-ray spectroscopy (EDX) showed these to be Fe-containing (Figure 1B), and electron diffraction (not shown) indicated a mixture of two phases – ferrihydrite-like (two broad diffraction rings characteristic of 2-line ferrihydrite [40]) and a second phase which was not uniquely identified (two broad diffraction rings at 0.20 and 0.13 nm). Under prolonged exposure to the electron beam the particles agglomerated further, developed in crystallinity and the diffraction pattern became dominated by this second phase: it was still not possible to confirm the identity of this alteration product but an Fe phosphate hydroxide provides the closest match to the observed lattice spacings (International Centre for Diffraction Data powder diffraction file 00-047-0413). Irradiation damage to poorly crystalline fine particles such as ferrihydrite [41] is well recognized, especially in the absence of supporting resin or tissue structure, so we considered this second phase to be artifactual. Hence the larger particles (10-20 nm) observed in the earlier work of Rudzki et al. [26] are, likely, an artifact of beam damage but the smaller particles are not. Moreover, the hydrolysis of the Fe(III) and the formation of these fine, disperse, ferrihydrite-like particles was not prevented by the presence of typical gastrointestinal low-molecular weight ligands. As a simplified and practical analogue to the above ferrihydrite-like particles we have developed ligand-modified (LM) Fe(III) poly oxo-hydroxides of similar size and structure [30]. This ligand-modified analogue remains well-dispersed upon aqueous suspension in the uptake medium used for cellular experiments (balanced salt solution: BSS) taking on an average hydrodynamic particle diameter of 10 nm (Figure 1C). 

**Figure 2:**
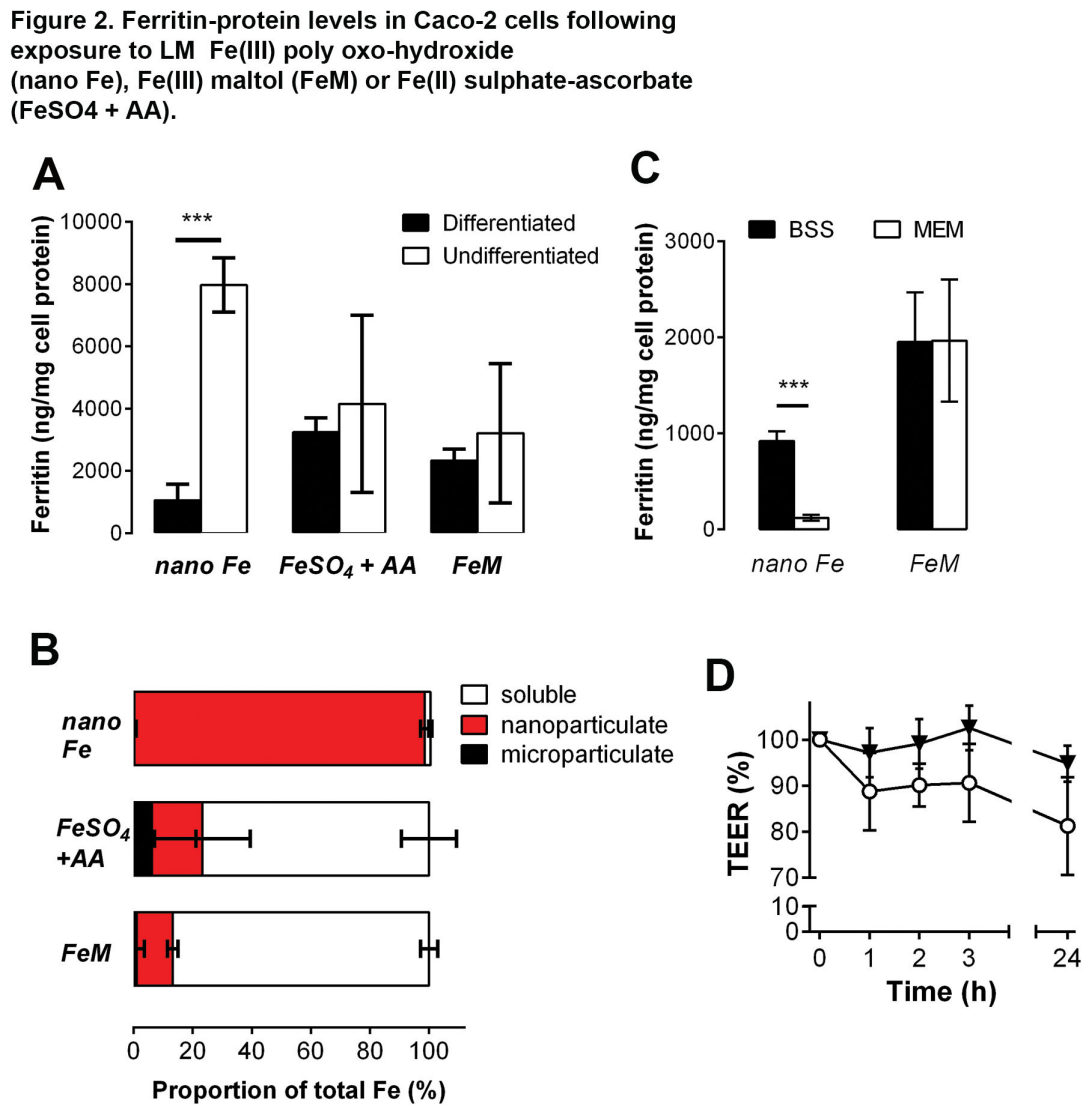
Ferritin-protein levels in Caco-2 cells following exposure to LM Fe(III) poly oxo-hydroxide (nano Fe), Fe(III) maltol (FeM) or Fe(II) sulphate-ascorbate (FeSO_4_ + AA). **A**, Ferritin-protein regulation in differentiated and undifferentiated cells. ***, *p*=0.0003. Cells were incubated for 1 h with 200 μM Fe plus a further 23 h in fresh, non-supplemented MEM to allow for ferritin formation. **B**, Phase distribution of Fe in the BSS uptake medium: i.e. fractional percentage of microparticulate (black bars), nanoparticulate (red bars) and soluble Fe (open bars) for each Fe material. Values are mean ± s.d. of 3 independent experiments. **C**, Effect of LM Fe(III) poly oxo-hydroxide particle dispersion (in BSS medium, closed bars) or agglomeration (in MEM medium, open bars) on ferritin-protein levels in differentiated cells: the LM Fe(III) poly oxo-hydroxide was dispersed in its nano-form (99 ± 2% nano) using BSS or agglomerated (97 ± 2% microparticulate) using MEM. Data are mean of 3 independent experiments (each experiment with 3 replicate wells). FeM: soluble iron control, Fe(III) maltol. ***, *p*=0.0002 for the comparison between BSS and MEM. **D**, TEER changes in differentiated Caco-2 cell monolayer at different time points during incubation with BSS supplemented with LM Fe(III) poly oxo-hydroxide (open circles) or non-supplemented BSS control (closed inverted triangles). Incubations were for 3 h with 200 μM Fe (measurements at 1, 2 & 3 h) plus a further 21 h in fresh, non-supplemented MEM (24-h). Values are expressed as a percentage of the initial measurement and are shown as mean ± s.d. of 3 independent experiments (each experiment with 3 replicate wells). Experimental points are connected with a solid line to aid visualization and not because a linear relationship is assumed between time and TEER measurement. Detailed methodology is available in the Methods Section and in Methods S1.

Here, ferritin-protein formation of epithelial cell cultures, as a measure of cellular iron utilisation, was assessed following a 24 h total incubation period that involved 1 h Fe exposure of cells in BSS followed by washing, change of medium, and a 23 h incubation in non-supplemented minimum essential medium (MEM) (i.e. with very low Fe content). We used soluble Fe, either as Fe(III) maltol [42] or Fe(II) sulphate-ascorbate as positive controls [31]. Initially, we used both differentiated and undifferentiated cells and iron utilization varied greatly for the LM Fe(III) poly oxo-hydroxide structures (undifferentiated > differentiated; *p*=0.0003) but not at all for the soluble Fe(III) (Figure 2A). Alkaline phosphatase levels were used as a marker for Caco-2 cell differentiation and the integrity of the cell monolayer was assured prior to the experiments (see Figure S1). The solid phase distribution, i.e. the partition of the Fe in the soluble, nanoparticulate (aquated) or microparticulate (agglomerated) fractions, largely confirmed the nanoparticulate nature of the LM Fe(III) poly oxo-hydroxide and the solubility of the Fe(III) maltol and Fe(II) sulphate-ascorbate (Figure 2B). Cellular uptake and utilization of the LM Fe(III) poly oxo-hydroxide required nano dispersion because purposeful agglomeration to microparticles (first hour in MEM, rather than in BSS, leading to 97 ± 2% microparticulate iron at 30 min) prevented iron utilization (Figure 2C). We confirmed that BSS did not affect monolayer integrity for the duration of the exposure (Figure 2D) although Fe(II) sulphate-ascorbate did [31], so further cellular work used Fe(III) maltol as the soluble control. 

**Figure 3:**
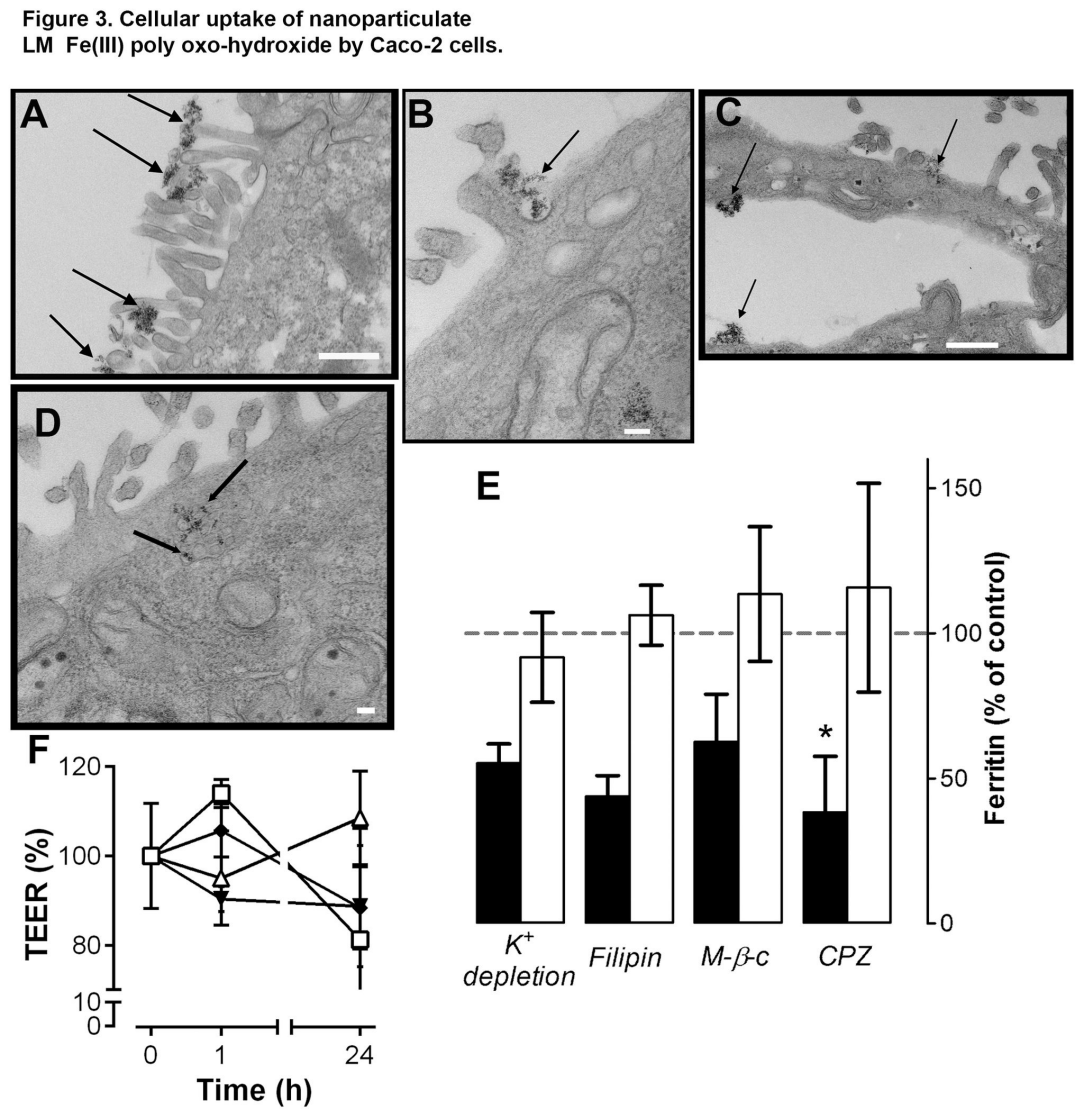
Cellular uptake of nanoparticulate LM Fe(III) poly oxo-hydroxide by Caco-2 cells. A, B, C, D TEM images showing differentiated Caco-2 cells incubated with LM Fe(III) poly oxo-hydroxide. **A**, arrows show particle clusters adhering to the cell membrane microvilli (scale bar, 500 nm). **B**, arrows indicate invagination on the cell membrane (scale bar, 100 nm). **C** and **D**, arrows indicate Fe accumulation inside the cell (scale bars: panel C, 500 nm and panel D, 100 nm). **E**, Effect of chemical inhibitors of endocytosis-related pathways on Fe utilization by differentiated Caco-2 cells. Data are shown as a percentage of the controls (without inhibitor) after a 1 h exposure to LM Fe(III) poly oxo-hydroxide (black bars) or Fe(III) maltol (open bars) co-incubated with either chlorpromazine (CPZ), potassium-free BSS (K^+^ depletion), filipin or methyl-β-cyclodextrin (M-β-c), plus 23 h in fresh non-supplemented MEM. Results are mean ± s.d. of 3 independent experiments with 3 replicate wells per experiment for chlorpromazine (*, *p*=0.02 relative to control) or triplicate wells of the same experiment for the remaining inhibitors. **F**, Change in TEER in the Caco-2 cell monolayer following a 1 h exposure to chlorpromazine (closed diamonds), K^+^-free BSS (open triangles), M-β-c (open squares) or non-supplemented BSS (closed inverted triangles) and with 23 h further incubation in fresh MEM (24 h total). Values are a percentage of the initial measurement (0 h) and are shown as mean ± s.d. as above. Experimental points are connected with a solid line to aid visualization and not because a linear relationship is assumed between time and TEER measurement. Detailed methodology is available in the Methods Section and in Methods S1.

Hence, the uptake and utilization of LM Fe(III) poly oxo-hydroxide required its dispersion in nanoparticulate form (Figure 2C) and the mechanism of acquisition appeared different to that of soluble Fe (Figure 2a). Next we investigated energy requirements for Fe uptake by comparing data at 37° C with those at low temperature (i.e. on ice). Fe utilization from Fe(III) maltol at low temperature was decreased by 95 % (95 ± 1; n=3) as expected, but from nanoparticulate LM Fe(III) poly oxo-hydroxide the reduction was only ca. 20 % (19 ± 13; n=3) (Figure S2). Apparent low temperature uptake of nanoparticles has been observed before [43,44] and is attributed to particle adhesion to the cell membrane. Adherent particles, that withstand washing, may be subsequently taken up by cells (and in this case utilized to form ferritin-protein, indicating that cells remained healthy) upon re-incubation in fresh medium at 37° C. Electron microscopy indicated cell surface adhesion of the nanoparticles and also showed that, generally, the cell membrane tended to induce loose agglomeration or aggregation of the particles into clusters up to 200 nm in diameter (Figure 3A). Thus we considered that Fe from the LM Fe(III) poly oxo-hydroxide nanoparticles was utilized following acquisition by endocytic uptake, as this is superior in undifferentiated versus differentiated Caco-2 cells [45], and cell surface adhesion is a requirement prior to uptake. Indeed further electron microscopy work revealed evidence of cell surface invagination (Figure 3B) with respect to the surface clusters of LM Fe(III) poly oxo-hydroxide and showed loose agglomerates of ≤ 200 nm in diameter within vesicles that we suggest are endosomes or lysosomes (Figure 3C, D). TEM images were also collected for control Caco-2 cells: i.e. cells incubated with non-supplemented BSS and cells incubated with BSS supplemented with the soluble Fe(III) maltol chelate and no discernible particulate Fe was observed (Figure S3). To determine whether nanoparticulate uptake contributed to cellular Fe utilization of the LM Fe(III) poly oxo-hydroxide we used an inhibitor of clathrin-mediated endocytosis, namely chlorpromazine, at a dose of 100 μM consistent with previous studies [32,33]. We confirmed that this significantly reduced (*p*=0.02) ferritin-protein formation in the cells exposed to LM Fe(III) poly oxo-hydroxide (Figure 3E), without significantly affecting monolayer integrity (Figure 3F), whilst no difference was observed for soluble iron uptake (Figure 3E, F). Further endocytic-inhibition studies with K^+^ depletion, filipin and methyl-β-cyclodextrin suggested the same picture: namely that Fe utilization appeared reduced albeit so only for LM Fe(III) poly oxo-hydroxide exposure and not for soluble Fe (Figure 3D). Interestingly, filipin and methyl-β-cyclodextrin inhibit caveolae-related rather than clathrin-mediated endocytosis so, consistent with previous nanoparticulate uptake work, there either appears to be a degree of redundancy/overlap between the uptake pathways [43] or precise uptake may be related to the size range of the nanoparticulate clusters formed on the cell membrane [46–49]. All cell monolayers appeared healthy under the microscope, and also without significant changes in TEER measurements, under the assay conditions used with K^+^-free BSS and methyl-β-cyclodextrin (Figure 3F), although no TEER assessment was made with filipin. 

### Lysosomal dissolution of ligand-modified Fe(III) poly oxo-hydroxide nanoparticles enables cellular utilization of iron

**Figure 4:**
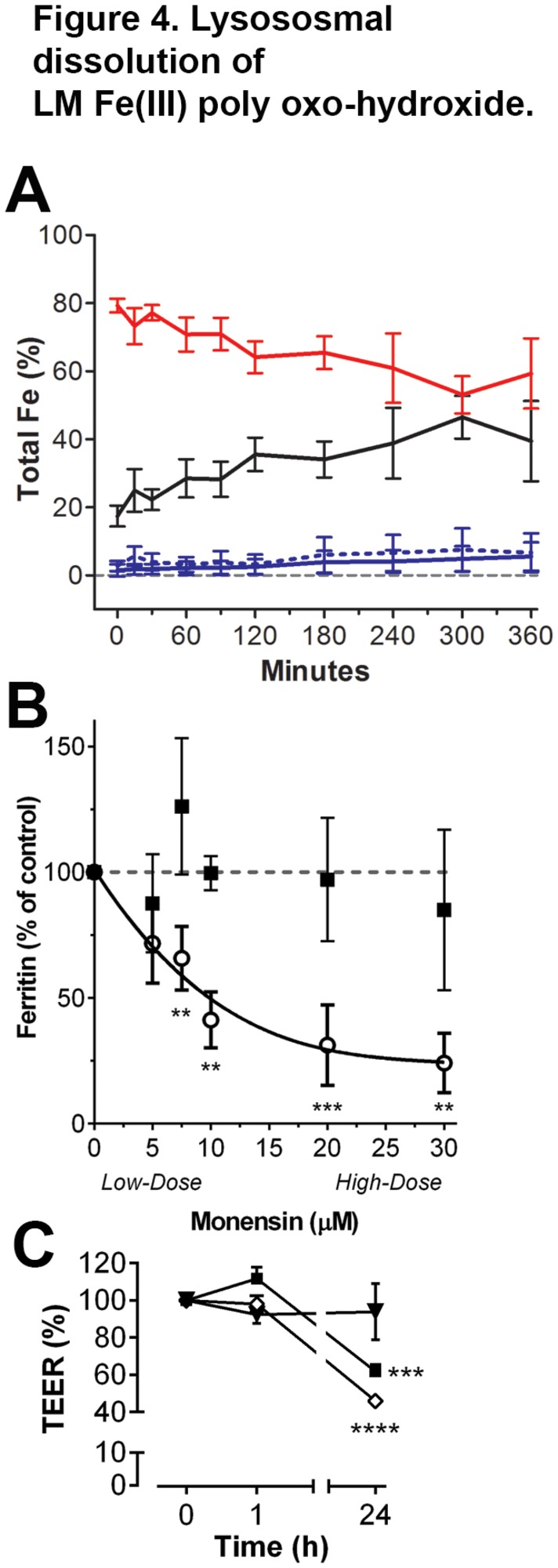
Lysososmal dissolution of LM Fe(III) poly oxo-hydroxide. **A**, Solubility in simulated lysosomal conditions at pH 5.0 with 10 mM citric acid and 0.15 M NaCl. Soluble Fe was measured by ICP-OES following 5 min ultrafiltration (3000 Da MWCO) for the LM Fe(III) poly oxo-hydroxide (black) and for un-modified Fe(III) poly oxo-hydroxide (solid blue). Nanoparticulate Fe was obtained from the Fe in the supernatant following centrifugation excluding the soluble (ultrafilterable) Fe, and is shown for LM Fe(III) poly oxo-hydroxide (red) and for un-modified Fe(III) poly oxo-hydroxide (dotted blue). Values are plotted as mean ± s.d. of 3 independent experiments (each experiment with 3 replicates). **B**, Effect of inhibition of lysosomal acidification using monensin on Fe utilization by differentiated Caco-2 cells. Data are shown as a percentage of the control (without monensin) at 24 h: i.e. 1 h exposure to 200 µM nanoparticulate LM Fe(III) poly oxo-hydroxide (open circles) or Fe(III) maltol (closed squares) ± 5-30 µM monensin followed by 23 h in non-supplemented MEM. Results are means ± s.d. of 3 independent experiments (each experiment with 3 replicate wells). **, *p*<0.005; ***, *p*<0.001 in relation to the soluble Fe control (Fe(III)maltol). **C**, Change in TEER in the Caco-2 cell monolayer following 1 h exposure to 10 μM monensin (closed squares), 30 μM monensin (open diamonds) or non-supplemented BSS control (closed inverted triangles) and with 23 h further incubation in fresh MEM (24 h in total). Values are expressed as a percentage of the initial measurement at the start of the exposure time (corresponding to 0 h) and are shown as mean ± s.d. of 2 independent experiments (each experiment with 3 replicate wells). Experimental points are connected with a solid line to aid visualization and not because a linear relationship is assumed between time and TEER measurement. ***, *p*=0.0003; ****, *p*<0.0001 in relation to the non-supplemented BSS control.

Overall, direct endocytosis of LM Fe(III) poly oxo-hydroxide nanoparticles by differentiated Caco-2 cells appears, chiefly, to explain uptake of Fe under these conditions. However, this Fe still needs to be released for cellular utilization (i.e. ferritin-protein synthesis in the current cell assay) and there would be insufficient intracellular (lysosomal) acidification for this alone to drive the release and cytosolic iron transit. Skotland et al. [50] developed a simplified endosomal/lysosomal solution that included 10 mM citrate and this chelator might, when aided by endosomal/lysosomal acidification, enable the breakdown and release of Fe from these fine, ligand-destabilized ferrihydrite-like particles. We showed that under these simplified endosomal/lysosomal conditions soluble Fe could be released within hours from the LM Fe(III) poly oxo-hydroxide (ferrihydrite) structures at pH 5.0 (Figure 4A) whereas, in the absence of ligand modification, the native Fe(III) poly oxo-hydroxide was poorly broken down and little soluble Fe was released (Figure 4A). This ‘destabilization’ role of low molecular weight ligands in the nano-structured Fe(III) poly oxo-hydroxide indicates a more likely physiological role for them in the gut lumen rather than, as originally proposed [19], as donors of Fe(III) to mucin. Next, using cell studies and monensin to inhibit endosomal/lysosomal acidification [39], we showed a monensin-dose-dependent reduction (i.e. Log_10_ [monensin] versus [ferritin-protein], r^2^=0.935, *p*<0.05) in Fe utilization by differentiated Caco-2 cells when exposed to LM Fe(III) poly oxo-hydroxide (Figure 4B), with statistically significant inhibition of ferritin formation at monensin doses ≥ 7.5 µM (*p*=0.002). Again, soluble Fe was unaffected. The monensin doses used here were equivalent to those reported previously in inhibition studies of endosomal/lysosomal acidification in Caco-2 cells [51,52] or other cell types [53,54], even though by 24 h some disruption of the monolayer was visible by TEER measurements particularly at the highest monensin dose (Figure 4C). Nonetheless, the monolayer was intact during the 1 hour cell exposure to Fe (Figure 4C) and, when examined microscopically, the cell monolayers appeared integral and healthy at doses of monensin up to 10 µM which still greatly inhibited ferritin-protein formation (Figure 4B), and there were similar protein levels in the presence and absence of the inhibitor (Figure S4). 

Finally, we confirmed that none of the inhibitors caused significant agglomeration of the nanoparticles in the supplemented uptake medium (Table S1). 

## Overall Discussion

The Caco-2 cell line has been extensively used for iron uptake studies [55–59] as following confluence it differentiates into a polarised cell monolayer that exhibits a phenotype similar to human small intestinal enterocytes [60,61].

Our data suggest that an endocytic, apical uptake mechanism exists in these differentiated Caco-2 cells for uptake of luminally hydrolysed (poly oxo-hydroxide) dietary Fe(III). This route apparently relies on endocytic uptake of small clusters of LM Fe(III) poly oxo-hydroxide nanoparticles which agglomerate at the cell membrane. Many cellular studies on nanoparticulate uptake and processing are challenged with issues of agglomeration and aggregation meaning that, finally, cells are exposed to micron-sized clusters which will be acquired and processed differently compared to genuinely disperse nanostructures [46,62,63]. Thus we paid considerable attention to developing a cell culture medium that mitigated against such pitfalls and retained appropriate dispersion of the particles prior to cell delivery [31]. The finding that these fine LM Fe(III) poly oxo-hydroxide nanoparticles then tend to agglomerate at the cell membrane must be interpreted with caution. It could be that the processing required to produce thin sections for electron microscopy induced clustering. However, small agglomerates of nanoparticles were observed in endosomal/lysosomal compartments within the cell and, so, the particles must have been taken up either as individual particles or clusters. Moreover, and importantly, we observed loose clusters almost always smaller than 200 nm diameter and it is at around this size that uptake mechanisms may switch between classical endocytosis and macropinocytosis [46,64]. There appears to be no mechanism of macropinocytosis in intestinal enterocytes, including differentiated Caco-2 cells, and phagocytosis has been rarely demonstrated [65,66]. The inhibitors used here were classical endocytic inhibitors and our findings suggest endocytosis of individual particles or membrane-induced small clusters. We accept that the specificity of K^+^-depletion to clathrin-mediated endocytosis and methyl-β-cyclodextrin to caveolin-dependent endocytosis in HuTu cells has been previously questioned [34]. However, chlorpromazine has been consistently shown to be highly specific for clathrin-mediated endocytosis in both HuTu and Caco-2 cells, providing comparable inhibition rates to siRNA approaches [34,67]. Filipin also appears specific to caveolin-dependent endocytosis [35,68]. Nonetheless, we do not, here, suggest dominance of one specific form of endocytosis for the uptake of LM Fe(III) poly oxo-hydroxide nanoparticles as further work may be required to conclude on this. 

Our observations on Fe utilization by Caco-2 cells, through endocytic uptake and lysosomal dissolution of nanoparticulate LM Fe(III) poly oxo-hydroxide, are still consistent with the established fact that DMT-1 is indispensable for mammalian Fe absorption and, therefore, survival [69]. DMT-1, as well as being the enterocyte Fe(II) apical transporter is probably also the export transporter of the lysosomal membrane [70–72]: i.e. the point from where endocytosed and then solubilized nanoparticulate Fe would join the common Fe pool. The elegant study of Gunshin et al. [69], utilizing an intestinal-specific DMT-1 knock-out model termed Slc11a2^*int/int*^ (which is a cross of Slc11a2^*flox/flox*^ mice with mice carrying a Villin-Cre transgene), proved essentiality of this transporter for intestinal non-haem iron absorption after birth [69]. However, the Slc11a2^*int/int*^ model has DMT1 knocked-out in the entire enterocyte and not solely from the apical membrane [69] and, therefore, this study did not eliminate DMT-1 functionality in both apical uptake and cytosolic processing of intracellular iron.A further point is that laboratory rodent diets poorly represent the human diet. Invariably the laboratory diet is rich in fibrous carbohydrate that is poorly digestible in the upper gastrointestinal tract and will bind Fe(III) thereby preventing mucosal access. Thus, Fe(II) sulphate is commonly supplemented into rodent laboratory chow, as for example in the RMH 3000 LabDiet (PMI Nutrition International) used by Gunshin et al. in the study referred to above [69]. In this case, the essentiality of an Fe(II) transporter would be favoured. 

Future *in vivo* rodent experiments could use a diet representing the more varied human diet for non-heme Fe (e.g., mixed Fe(II)-Fe(III) salts that undergo gastric dissolution, plus ferritin-protein) and then establish, in the Slc11a2^*int/int*^ mouse, whether or not there is enterocyte lysosomal accumulation of Fe (i.e. initial apical uptake due to a non DMT-1 pathway for the hydrolysed Fe(III) and possibly ferritin-protein fractions, but accumulation due to export failure with lack of lysosomal DMT-1). Such a diet fed to Slc11a2^*int/int*^ mice will delineate the essential role of DMT-1 for apical iron uptake versus lysosomal iron efflux. Indeed, Kalgaonkar and Lonnerdal [3] have speculated that dietary ferritin Fe, which contains subunits of ferrihydrite similar in structure to the LM Fe(III) poly oxo-hydroxide nanoparticles used herein [73], may not all be dissolved under post-prandial gastric conditions, and could be absorbed whole by duodenal enterocytes and subsequently broken down intra-lysosomally. Moreover, elsewhere in mammalian Fe physiology, the lysosome is a safe reservoir for catabolism of Fe-rich species, whether it be ferritin-protein, red blood cells or transferrin turnover [71,74]. Free Fe is potentially redox active and thus toxic to cells. So the concept that cells lining the gut are protected from redox damage (Fe(III) ↔ Fe(II) + e^-^) due to luminal nanoparticulate LM Fe(III) poly oxo-hydroxide formation, which is only available for breakdown in the safest compartment of the cell, i.e. the endosome/lysosome, is consistent with other aspects of Fe metabolism. Finally, of particular note, is a paper by Theil and colleagues [75], published during preparation of this manuscript, where they show that the Fe derived from dietary ferritin-protein (i.e. protein-encapsulated ferrihydrite-like nanostructures) is absorbed differently in rats and humans to that of DMT-1-dependent Fe(II). Moreover, their findings invoke an epithelial endocytic process, which we believe we detail here: i.e. the fraction of dietary iron derived from ferritin-protein ingestion could lose its protein shell but maintain its ferrihydrite-like structure following gastrointestinal digestion and ‘ride’ the endocytic mechanisms that exists for endogenously formed LM Fe(III) poly oxo-hydroxide. Ultimately, the two forms may look very similar, being 2-5 nm diameter, destabilized ferrihydrite [73] and thus, readily solubilized by the enterocyte endosome/lysosome. 

Our preliminary data in duodenal epithelial cells (HuTu-80 cell line) using siRNA targeting DMT1 further supports a pathway independent of DMT1 and Dcyt B for the intestinal uptake of LM Fe(III) poly oxo-hydroxide and, thus, not involving reduction of Fe(III) to Fe(II) (Figure S5). 

We have demonstrated the existence of a nanoparticulate ferrihydrite-like phase in a model of luminally hydrolysed Fe(III) and, using a synthetic nanoparticulate ligand-modified ferrihydrite as an analogue, we have shown the existence of an endocytic pathway of Fe uptake by Caco-2 cells, a gut epithelial cell model, followed by lysosomal dissolution of nanoparticulate LM Fe(III) poly oxo-hydroxide which is consistent with *in vivo* observations elsewhere [75]. 

These *in vitro* mechanistic findings now need to be replicated by *in vivo* data for physiological relevance, and, it is noteworthy that, when supplemented into the diet of iron deficient rats, these LM Fe(III) poly oxo-hydroxide structures improved haemoglobin levels in line with Fe(II) sulphate supplementation [Pereira, Latunde-Dada and Powell unpublished observations]. 

## Supporting Information

Methods S1 (DOCX)

Table S1 **Iron solid-phase distribution of 200 µM Fe as LM Fe(III) poly oxo-hydroxide (nano Fe) and Fe(III) maltol (FeM) co-incubated with different chemical inhibitors in the BSS uptake medium.**
(DOCX)

Figure S1 **Caco-2 cell growth and differentiation parameters in monolayers grown for 21 days.** **A**, cell protein (open circles) and transepithelial electrical resistance (TEER, closed squares). Data shown are mean ± s.d. of n=3 independent experiments (each experiment with 12 replicate wells). **B**, alkaline phosphatase activity (closed triangles). Data shown are mean ± s.d. of n=3 independent experiments (each experiment with 3 replicate wells). Under these culture conditions the cell monolayer is fully confluent at day 5 post-seeding and differentiated at day 10 post-seeding in accordance with reference values reported in the literature [76,77]. .
(TIF)

Figure S2 **Effect of low temperature incubation on ferritin-protein levels in Caco-2 cells following exposure to LM Fe(III) poly oxo-hydroxide (nano Fe) or Fe(III) maltol (FeM).** Cells were incubated for 1 h in BSS at 37°C (closed bars) or 4°C (open bars) supplemented with 200 µM Fe plus a further 23 h in fresh, non-supplemented MEM to allow for ferritin formation. Data are mean (± s.d.) of 3 replicate wells within one experiment. **, *p*=0.001.
(TIF)

Figure S3 **TEM image showing differentiated Caco-2 cells incubated at 37° C for 3 h with (A) non-supplemented control media (i.e. balanced salt solution, BSS), (**B**) control BSS supplemented with maltol alone and (**C**) BSS supplemented with 500 µM Fe as the soluble Fe(III) maltol chelate.** Scale bar, 500 nm.
(TIF)

Figure S4 **Effect of monensin on cellular protein levels in Caco-2 monolayers.** Cells were incubated for 1 h in BSS supplemented with different concentrations of monensin plus a further 23 h in fresh, non-supplemented MEM. Box and whisker plots show median, minimum and maximum for n=6 independent experiments. The red solid line represents the mean cellular protein for all experiments and the dotted red lines represent the corresponding 95% confidence interval. No statistical differences were found for any monensin concentration in relation to control cells incubated without monensin (0 µM).
(TIF)

Figure S5 **Effect of siRNA targeting of DMT1 (closed bars) and Dcyt B (open bars) on iron uptake in HuTu cells.** HuTu cells were transfected with siRNAs and, after 3 days, the cells were incubated for 1 h in BSS supplemented with 10 µM Fe as soluble Fe(II) (ferrous sulphate) or LM Fe(III) poly oxo-hydroxide (nano Fe) plus a further 23 h in fresh, non-supplemented MEM to allow for ferritin formation. Pattern bars indicate scrambled-transfected cells. Data are mean (± s.d.) of 3 replicate wells within one experiment. ****, *p*<0.0001.
(TIF)
