# Correction: Caco-2 Cell Acquisition of Dietary Iron(III) Invokes a Nanoparticulate Endocytic Pathway

**DOI:** 10.1371/journal.pone.0119747

**Published:** 2015-03-20

**Authors:** The PLOS ONE Staff

There is an error in the first sentence of the “Cellular uptake studies” subsection of the Materials and Methods. The correct sentence is: To avoid aggregation/agglomeration of the nanoparticulate iron, the medium for cellular uptake consisted of a balanced salt solution (BSS) containing 130 mM NaCl, 10 mM KCl, 1 mM MgSO4, 5 mM Glucose and 1.8 mM CaCl_2_ in 10 mM PIPES buffer (pH 7.4).

## References

[1] Pereira DIA, Mergler BI, Faria N, Bruggraber SFA, Aslam MF, Poots LK, et al (2013) Caco-2 Cell Acquisition of Dietary Iron(III) Invokes a Nanoparticulate Endocytic Pathway. PLoS ONE 8(11): e81250 doi:10.1371/journal.pone.0081250 24278403 10.1371/journal.pone.0081250 PMC3836913

