# Supplementary material for: Caco-2 Cell Acquisition of Dietary Iron(III) Invokes a Nanoparticulate Endocytic Pathway
Source: PLoS One. 2013 Nov 21;8(11):e81250. doi: 10.1371/journal.pone.0081250 (PMC3836913; doi:10.1371/journal.pone.0081250)
Supplement: Methods S1 — (DOCX) [file pone.0081250.s001.docx]

**Caco-2 cell acquisition of dietary iron(III) invokes a nanoparticulate endocytic pathway.**

Dora IA Pereira, Bianca I Mergler, Nuno Faria, Sylvaine FA Bruggraber, Mohamad F Aslam, Lynsey K Poots, Laura Prassmayer, Bo Lönnerdal, Andy P Brown, Jonathan J Powell

**Supplementary Information**

**Supplementary Methods S1:**

**Reagents.** All chemicals and reagents were obtained from Sigma-Aldrich (Dorset, UK) with a minimum of 99% purity unless otherwise stated. All material suspensions were prepared in 18.2 MΩ·cm^-1^ ultrapure water.

**Chemical characterization of the Fe materials.**

Transmission Electron Microscopy (TEM) after simulated digestion. The Fe suspensions used for TEM analysis were derived from exposing Fe(III) chloride to an *in house* assay designed to mimic hydrolysis of Fe(III) under conditions of digestion. Briefly, Fe(III) chloride was added ([Fe]=3.5mM) to simulated gastric fluid containing NaCl (0.15 M), mucin (2 g/L), HCl (0.15 M), and a low molecular weight ligand mixture (described below), at pH 1.2 (1.1-1.3). This suspension was incubated at 37^o^ C for 30 min in a shaking water bath (150 rpm). Mucin (porcine mucin type II) was used at physiological levels [[1](#_ENREF_1),[2](#_ENREF_2)]. The low molecular weight ligand mixture was based on physiological levels of amino acids, organic acids and ascorbic acid [[3](#_ENREF_3),[4](#_ENREF_4),[5](#_ENREF_5),[6](#_ENREF_6)] and contained (in mM): lactate (0.311), glutamate (0.14), lysine (0.096), histidine (0.084), citric acid (0.255), acetate (12), glutamine (1.4), arginine (0.19), aspartic acid (0.13), and ascorbic acid (0.015). Following gastric dissolution, the pH of the resulting suspension was neutralized to pH 6.5-7.1 with 50 mM Na_2_CO_3_/NaHCO_3_ buffer (pH 9.5) containing NaCl (0.15 M), mucin (2 g/L), and the low molecular weight ligand mixture as above. This simulated intestinal fluid mixture was then incubated at 37^o^C for 30 min in a shaking water bath (150 rpm). The final Fe concentration was ca. 1 mM. Samples of this intestinal mixture were then drop-cast directly onto holey carbon TEM support film (on a Cu-grid) and air-dried prior to TEM analysis. TEM investigations were carried out using a Philips CM200 FEG-TEM operating at 197 keV and fitted with a Gatan Imaging Filter (GIF 200) and Oxford Instruments UTW ISIS X-ray detector (EDS).

Lysosomal dissolution assay. The solubility of LM Fe(III) poly oxo-hydroxide and un-modified Fe(III) poly oxo-hydroxide (i.e. standard synthetic ferrihydrite) was determined at pH 5.0 ± 0.1 in a 10 mM citric acid, 0.15 M NaCl solution. The Fe material was suspended in the assay solution at an Fe concentration of ca. 1 mM and incubated for 360 min at room temperature. The pH was automatically maintained at 5.0 using 1 M NaOH and 1 M HCl with an autotitrator (Metrohm, Malvern Instruments, U.K.). Samples were collected at different time points throughout the incubation period for analysis of Fe content. The assay was performed in triplicate. The soluble and nanoparticulate Fe fractions were determined as explained below. Results were compared to similarly prepared Fe(III) poly oxo-hydroxide without ligand modification.

Particle size and phase distribution. The hydrodynamic particle size of the nanoparticulate LM Fe(III) poly oxo-hydroxide material was determined by Dynamic Light Scattering (DLS) and the non-aquated primary particle size by Transmission Electron Microscopy (TEM). DLS was performed using a Zetasizer, Nanoseries Nano ZS (Malvern Instruments Ltd., Worcestershire, UK) to measure the size of particles in suspension in the medium used for the cellular assays immediately after centrifugation (10,000  *xg*, 5 min). Centrifugation was used prior to DLS measurements to remove and quantitate any sedimenting large agglomerates that would interfere with the measurements. Fractionation of the Fe into percentages of nanoparticulate, microparticulate and soluble Fe, for each of the Fe materials in the medium used for the cellular assays was achieved by centrifugation and ultrafiltration. Aquated samples were centrifuged (10,000 *xg*, 5 min) and the sediment considered as the microparticulate fraction. In order to isolate the soluble Fe and to distinguish it from nanoparticulate Fe the supernatant was further ultrafiltered (3,000 Da MWCO; 10,000 *xg*, 10 min). The Fe content of all fractions (i.e. total, supernatant and ultrafiltrate) was determined by inductively-coupled plasma optical emission spectrometry (ICP-OES JY 2000, Horiba Jobin Yvon Ltd., Stanmore, UK) at 259.94 nm. Prior to analysis the samples were diluted in 0.5% HNO_3_ and the Fe content determined against a series of matrix-matched standards in the range 0-1000 ppb. The Fe content in these fractions was expressed as percentage ± s.d. in relation to total Fe content as follows:

[(%) Fe microparticulate] = [(total Fe – Fe supernatant)/total Fe] x 100

[(%) Fe nanoparticulate] = [(Fe supernatant – Fe ultrafiltrate)/total Fe] x 100

[(%) Fe soluble] = [(Fe ultrafiltrate)/total Fe] x 100

**Caco-2 cell culture.** Human adenocarcinoma (Caco-2) cells, obtained from the ATCC (LGC standards, Middlesex, United Kingdom) were grown at 37^o^ C in an atmosphere of 5 % CO_2_ and 95 % air at a relative humidity of approximately 95% and used for experiments at passages 24 – 50. Cells were maintained in T-75 flasks using Minimum Essential Medium (MEM; PAA Laboratories, Yeovil, UK) supplemented with 10 % foetal bovine serum (FBS “Gold”, PAA Laboratories), 1 % penicillin/streptomycin and 1 % fungizone (Invitrogen, Paisley, UK). The growth medium was changed every 2-3 days. Cells were passaged at 70- 80 % confluence using trypsin 0.25 %(w/v) in 1 mM ethylenediaminetetraacetic acid (EDTA) solution.

**Transepithelial electrical resistance (TEER).** TEER measurements were carried out to determine if the chemical inhibitors used in the uptake studies had a negative impact on the integrity of the cell monolayer (an early indication of toxicity). Cells were seeded onto polycarbonate 12-well Transwell inserts (Greiner, Stonehouse, UK) with a mean pore size of 0.4 μm. Caco-2 cells were maintained under the culture conditions described above and used at 21 days post-seeding. TEER was assessed prior to the experiments to verify the presence of an intact monolayer with values above 200 Ω·cm^2^ [[7](#_ENREF_7),[8](#_ENREF_8),[9](#_ENREF_9)]. The volt-ohm meter EVOM2 (WPI, Sarasota, USA) was calibrated and the electrode STX2 was equilibrated in pre-warmed medium before each measurement. Prior to the experiments, growth medium was aspirated and exchanged for serum-free MEM in both the apical and basolateral chambers. After a minimum of 2 h, MEM was aspirated from the apical compartment and the insert was washed once with pre-warmed DPBS. The cell monolayers were then incubated for 1 h with the non-supplemented uptake medium or the uptake medium supplemented with the inhibitors. Baseline TEER was measured ca. 5 min after addition of the test medium, which was considered as time zero and then TEER was measured at the required time points thereafter. The resistance was recorded for each well individually and the results were displayed as the average of triplicate wells.

**Caco-2 cell differentiation.** Alkaline phosphatase was determined using the Abnova (Heidelberg, Germany) assay kit according to manufacturer’s protocol.

**TEM of cell monolayers.** Electron microscopy was used to visualize nanoparticulate Fe uptake by the Caco-2 cells. Cells were seeded onto 6-well cell culture plates and used at 12 days post-seeding as before, and were then incubated for 3 h at 37°C with BSS supplemented with the LM Fe(III) poly oxo-hydroxide (500 µM Fe). Following incubation, the cells were washed three times with PBS-EDTA (2 mM) to remove any loosely bound Fe and washed once in 0.15 M NaCl prior to being fixed for 2 h in 4 % (w/v) glutaraldehyde. Cells were then gently scraped, collected in 15 mL tubes and pelleted by centrifugation at 1500 x*g* for 10 min. The pellet was then rinsed in 0.9 % (w/v) saline and stored in the fridge at 4° C until further treatment. Samples were subsequently dehydrated, embedded in resin and bulk stained for ultrastructural imaging following standard protocols [[10](#_ENREF_10),[11](#_ENREF_11)]. Sections of approximately 100 nm thickness were cut using a Leica Ultracut UCT ultra microtome and collected on 300-mesh copper grids, prior to visualization using a TEM (FEI-Philips CM100) operating at 120 kV in bright field mode. TEM images were also collected for control Caco-2 cells: i.e. cells incubated with non-supplemented BSS and cells incubated with BSS supplemented with the soluble Fe(III) maltol chelate (Data shown in Supplementary Figure 1).

**siRNA transfection.** HuTu80 cells (ATCC) were cultured at 37^o^ C in an atmosphere of 5 % CO_2_ and 95 % air at a relative humidity of approximately 95%. We used the HuTu80 duodenal epithelial cell line for the siRNA work as Caco-2 cells are considerably resistant to transfection and, even when achieved, transfection efficiency is very variable [[12](#_ENREF_12),[13](#_ENREF_13),[14](#_ENREF_14)]. Cells were maintained in Dulbecco’s Modified Eagle Medium (DMEM) supplemented with 10% FBS, 100 kU/L of penicillin and 100 mg/L streptomycin. Small interfering RNA oligonucleotides were purchased from Invitrogen Life Technologies: *Silencer*® Select Negative Control No. 1 (4390843, scramble), *Silencer*® Select siRNA targeting Dcyt B (s230761) and *Silencer*® Select siRNA targeting DMT1 (S9708). Hutu80 cells at 50% confluence were transfected with siRNAs (10nM) using RiboCellin transfection reagent (BioCellChallenge, France), following protocols provided by the manufacturer. Iron uptake studies were conducted 72 h after transfection. Cells were deprived of serum for 4 hours prior to uptake studies. Cells were then incubated for 1 h with 10 µM Fe as nano Fe or soluble Fe in BSS, followed by incubation in serum-free MEM for an additional 23 h to allow for ferritin-protein formation. Cells were lysed and analysed for ferritin and protein content as described in the main Methods section.

**Supplementary References**

1. Allen A, Flemstrom G (2005) Gastroduodenal mucus bicarbonate barrier: protection against acid and pepsin. American Journal of Physiology-Cell Physiology 288: C1-C19.

2. Atuma C, Strugala V, Allen A, Holm L (2001) The adherent gastrointestinal mucus gel layer: thickness and physical state in vivo. American Journal of Physiology-Gastrointestinal and Liver Physiology 280: G922-G929.

3. Atanasova BD, Li ACY, Bjarnason I, Tzatchev KN, Simpson RJ (2005) Duodenal ascorbate and ferric reductase in human iron deficiency. American Journal of Clinical Nutrition 81: 130-133.

4. Zhang ZW, Patchett SE, Perrett D, Katelaris PH, Domizio P, et al. (1998) The relation between gastric vitamin C concentrations, mucosal histology, and CagA seropositivity in the human stomach. Gut 43: 322-326.

5. Fraser AG, Woollard GA (1999) Gastric juice ascorbic acid is related to Helicobacter pylori infection but not ethnicity. Journal of Gastroenterology and Hepatology 14: 1070-1073.

6. Powell JJ, Whitehead MW, Ainley CC, Kendall MD, Nicholson JK, et al. (1999) Dietary minerals in the gastrointestinal tract: hydroxypolymerisation of aluminium is regulated by luminal mucins. J Inorg Biochem 75: 167-180.

7. Van Gelder J, Witvrouw M, Pannecouque C, Henson G, Bridger G, et al. (1999) Evaluation of the potential of ion pair formation to improve the oral absorption of two potent antiviral compounds, AMD3100 and PMPA. International Journal of Pharmaceutics 186: 127-136.

8. MacCallum A, Hardy SP, Everest PH (2005) Campylobacter jejuni inhibits the absorptive transport functions of Caco-2 cells and disrupts cellular tight junctions. Microbiology 151: 2451-2458.

9. Sabboh-Jourdan H, Valla F, Epriliati I, Gidley MJ (2011) Organic acid bioavailability from banana and sweet potato using an in vitro digestion and Caco-2 cell model. Eur J Nutr 50: 31-40.

10. Kuo J (2007) Electron microscopy : methods and protocols. Totowa, N.J.: Humana Press. xv, 608 p. p.

11. Motskin M, Wright DM, Muller K, Kyle N, Gard TG, et al. (2009) Hydroxyapatite nano and microparticles: correlation of particle properties with cytotoxicity and biostability. Biomaterials 30: 3307-3317.

12. Yu CF, Sanders MA, Basson MD (2000) Human caco-2 motility redistributes FAK and paxillin and activates p38 MAPK in a matrix-dependent manner. Am J Physiol Gastrointest Liver Physiol 278: G952-966.

13. Sanders MA, Basson MD (2005) p130cas but not paxillin is essential for Caco-2 intestinal epithelial cell spreading and migration on collagen IV. J Biol Chem 280: 23516-23522.

14. Uduehi AN, Moss SH, Nuttall J, Pouton CW (1999) Cationic lipid-mediated transfection of differentiated Caco-2 cells: a filter culture model of gene delivery to a polarized epithelium. Pharm Res 16: 1805-1811.
