# Supplementary material for: Caco-2 Cell Acquisition of Dietary Iron(III) Invokes a Nanoparticulate Endocytic Pathway
Source: PLoS One. 2013 Nov 21;8(11):e81250. doi: 10.1371/journal.pone.0081250 (PMC3836913; doi:10.1371/journal.pone.0081250)
Supplement: Table S1 — Iron solid-phase distribution of 200 µM Fe as LM Fe(III) poly oxo-hydroxide (nano Fe) and Fe(III) maltol (FeM) co-incubated with different chemical inhibitors in the BSS uptake medium. (DOCX) [file pone.0081250.s002.docx]

**Caco-2 cell acquisition of dietary iron(III) invokes a nanoparticulate endocytic pathway.**

*Dora IA Pereira, Bianca I Mergler, Nuno Faria, Sylvaine FA Bruggraber, Mohamad F Aslam, Lynsey K Poots, Laura Prassmayer, Bo Lönnerdal, Andy P Brown, Jonathan J Powell*

**Supplementary Information**

**Table S1. Iron solid-phase distribution of 200 µM Fe as LM Fe(III) poly oxo-hydroxide (nano Fe) and Fe(III) maltol (FeM) co-incubated with different chemical inhibitors in the BSS uptake medium.** Data represent percentage of the total Fe where soluble, nanoparticulate (nano) and microparticulate (micro) Fe fractions were determined as described in the Materials and Methods Section. Data are shown as mean (s.d.) of 3 independent experiments for chlorpromazine and monensin (each experiment with 3 replicate wells), and as mean of one experiment (3 replicate wells) for the remaining inhibitors.

|  | **nano Fe** | | | **FeM** | | |
| --- | --- | --- | --- | --- | --- | --- |
|  | **soluble** | **nano** | **micro** | **soluble** | **nano** | **micro** |
|  | **(%)** | | | **(%)** | | |
| Chlorpromazine | 1.4 (1.2) | 107.0* (16.8) | 2.6 (4.4) | 90.8 (5.2) | 10.7 (6.1) | 0* (2.0) |
| Monensin | 0.3 (0.4) | 97.5  (5.3) | 2.9 (5.0) | 93.7 (2.8) | 6.7  (3.4) | 0* (1.6) |
| K^+^ depletion | 2. 7 | 103.9* | 0* | 95.3 | 4.6 | 0.04 |
| Filipin | 2.3 | 95.4 | 2.3 | 85.6 | 13.7 | 0.8 |
| Methyl-β-cyclodextrin | 1.2 | 102.3* | 0* | 92.3 | 9.7 | 0* |

*Values >100 % lie within the expected range of the compound analytical uncertainty

(typically this is below 4 % but may reach ~ 8%); values below the limit of detection are shown as 0.
