# Supplementary figures and images for: Caco-2 Cell Acquisition of Dietary Iron(III) Invokes a Nanoparticulate Endocytic Pathway
Source: PLoS One. 2013 Nov 21;8(11):e81250. doi: 10.1371/journal.pone.0081250 (PMC3836913; doi:10.1371/journal.pone.0081250)

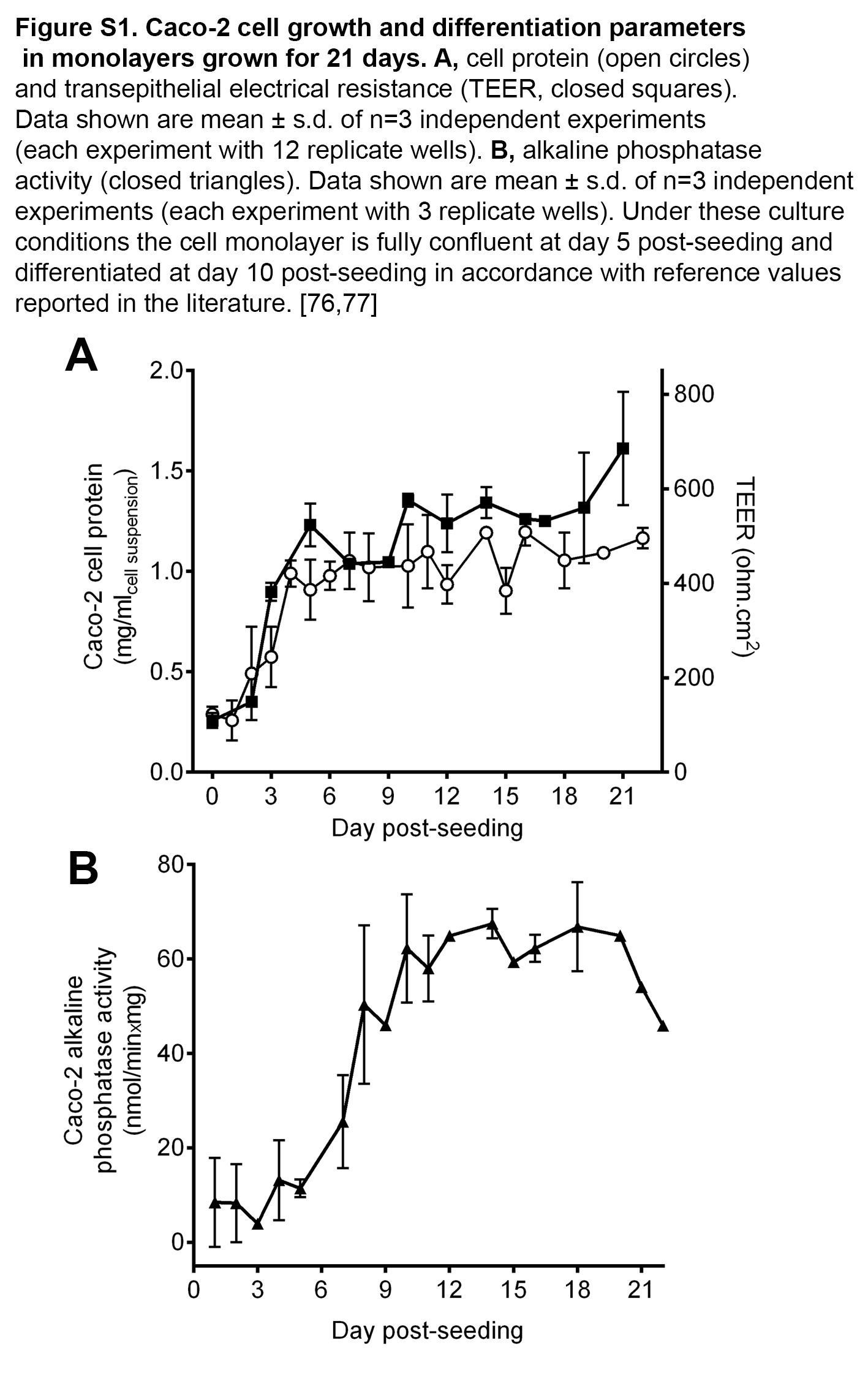

Supplement: Figure S1 — Caco-2 cell growth and differentiation parameters in monolayers grown for 21 days. A, cell protein (open circles) and transepithelial electrical resistance (TEER, closed squares). Data shown are mean ± s.d. of n=3 independent experiments (each experiment with 12 replicate wells). B, alkaline phosphatase activity (closed triangles). Data shown are mean ± s.d. of n=3 independent experiments (each experiment with 3 replicate wells). Under these culture conditions the cell monolayer is fully confluent at day 5 post-seeding and differentiated at day 10 post-seeding in accordance with reference values reported in the literature [76,77]. . (TIF) [file pone.0081250.s003.tif]

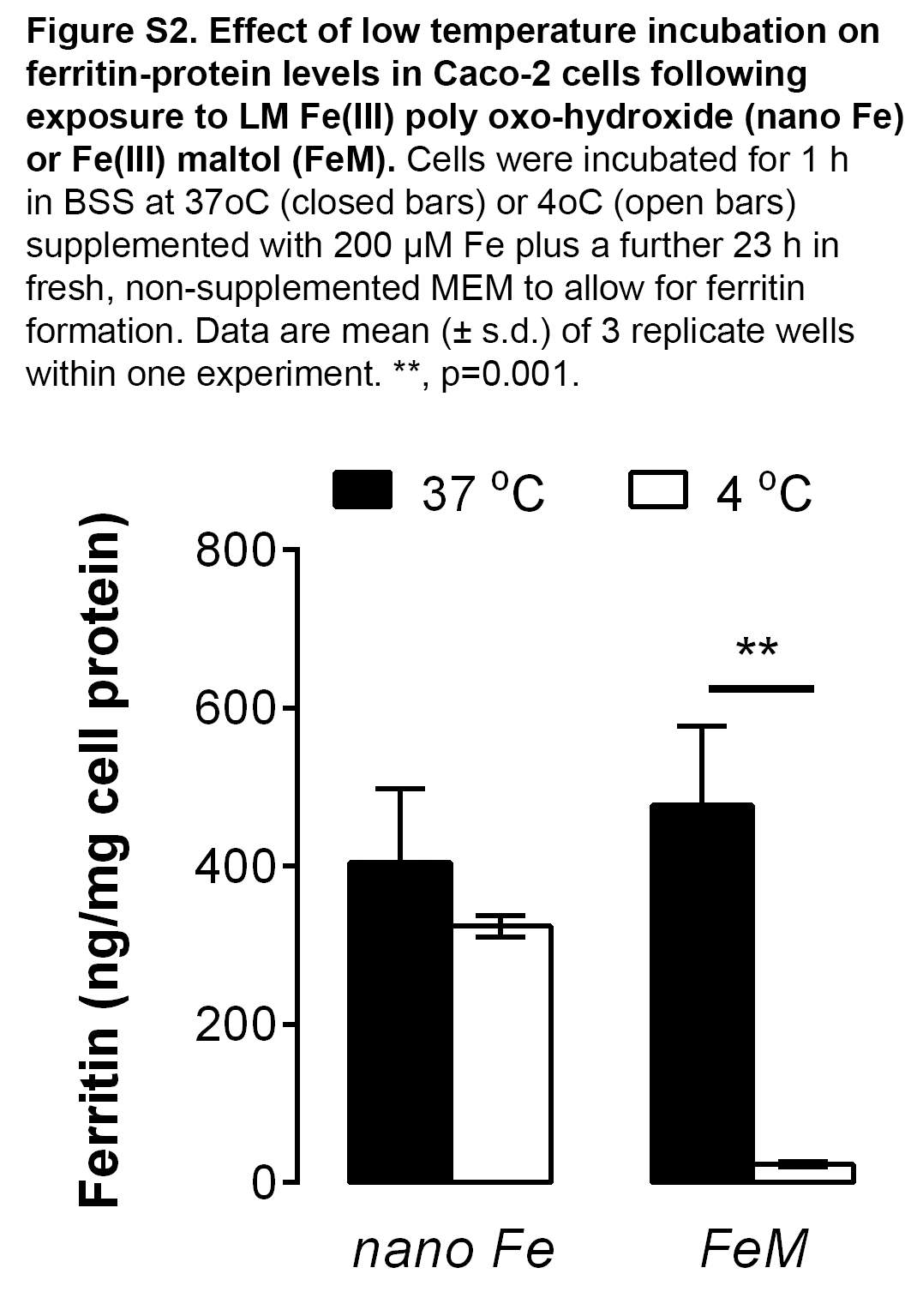

Supplement: Figure S2 — Effect of low temperature incubation on ferritin-protein levels in Caco-2 cells following exposure to LM Fe(III) poly oxo-hydroxide (nano Fe) or Fe(III) maltol (FeM). Cells were incubated for 1 h in BSS at 37°C (closed bars) or 4°C (open bars) supplemented with 200 µM Fe plus a further 23 h in fresh, non-supplemented MEM to allow for ferritin formation. Data are mean (± s.d.) of 3 replicate wells within one experiment. **, p=0.001. (TIF) [file pone.0081250.s004.tif]

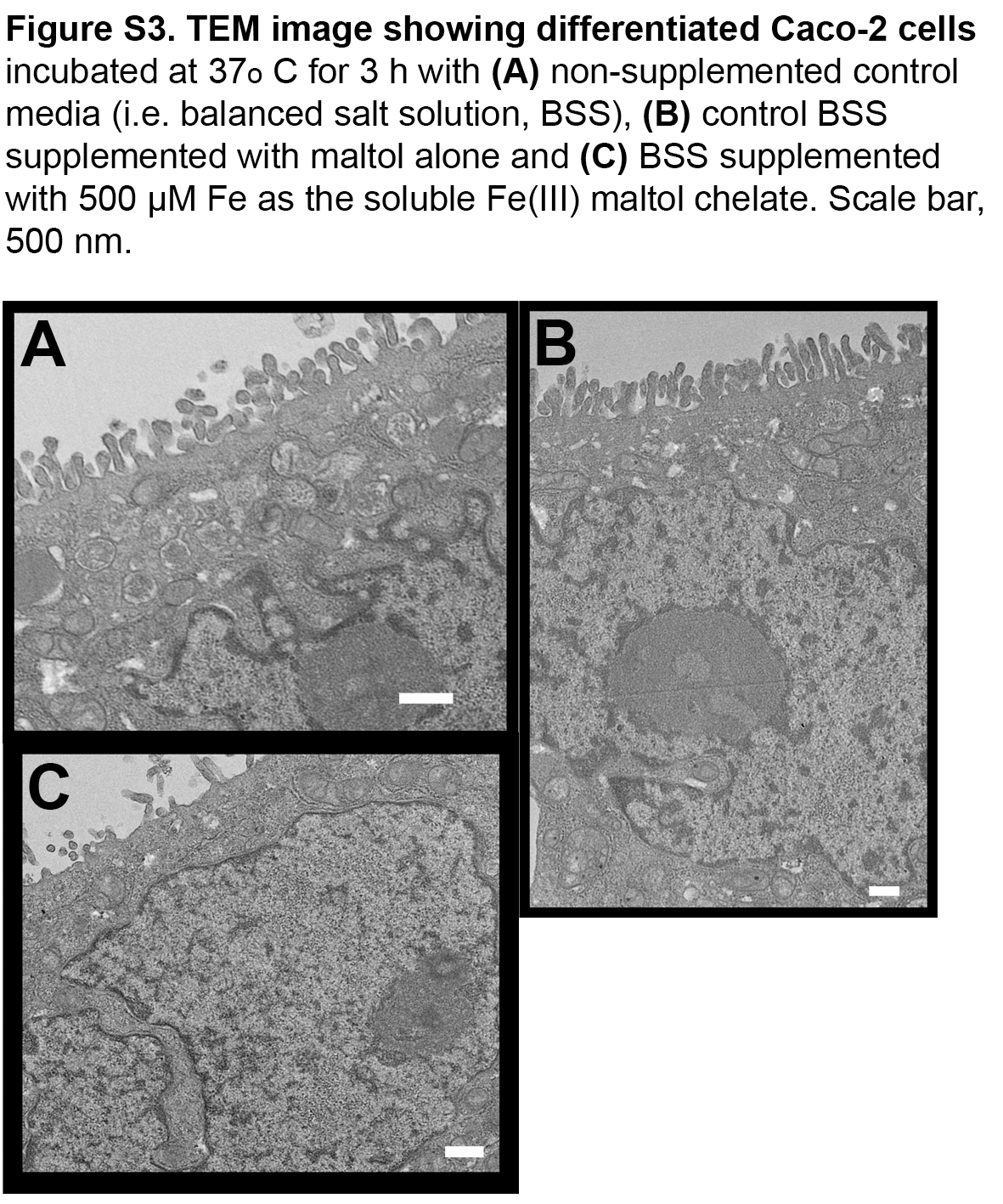

Supplement: Figure S3 — TEM image showing differentiated Caco-2 cells incubated at 37° C for 3 h with (A) non-supplemented control media (i.e. balanced salt solution, BSS), (B) control BSS supplemented with maltol alone and (C) BSS supplemented with 500 µM Fe as the soluble Fe(III) maltol chelate. Scale bar, 500 nm. (TIF) [file pone.0081250.s005.tif]

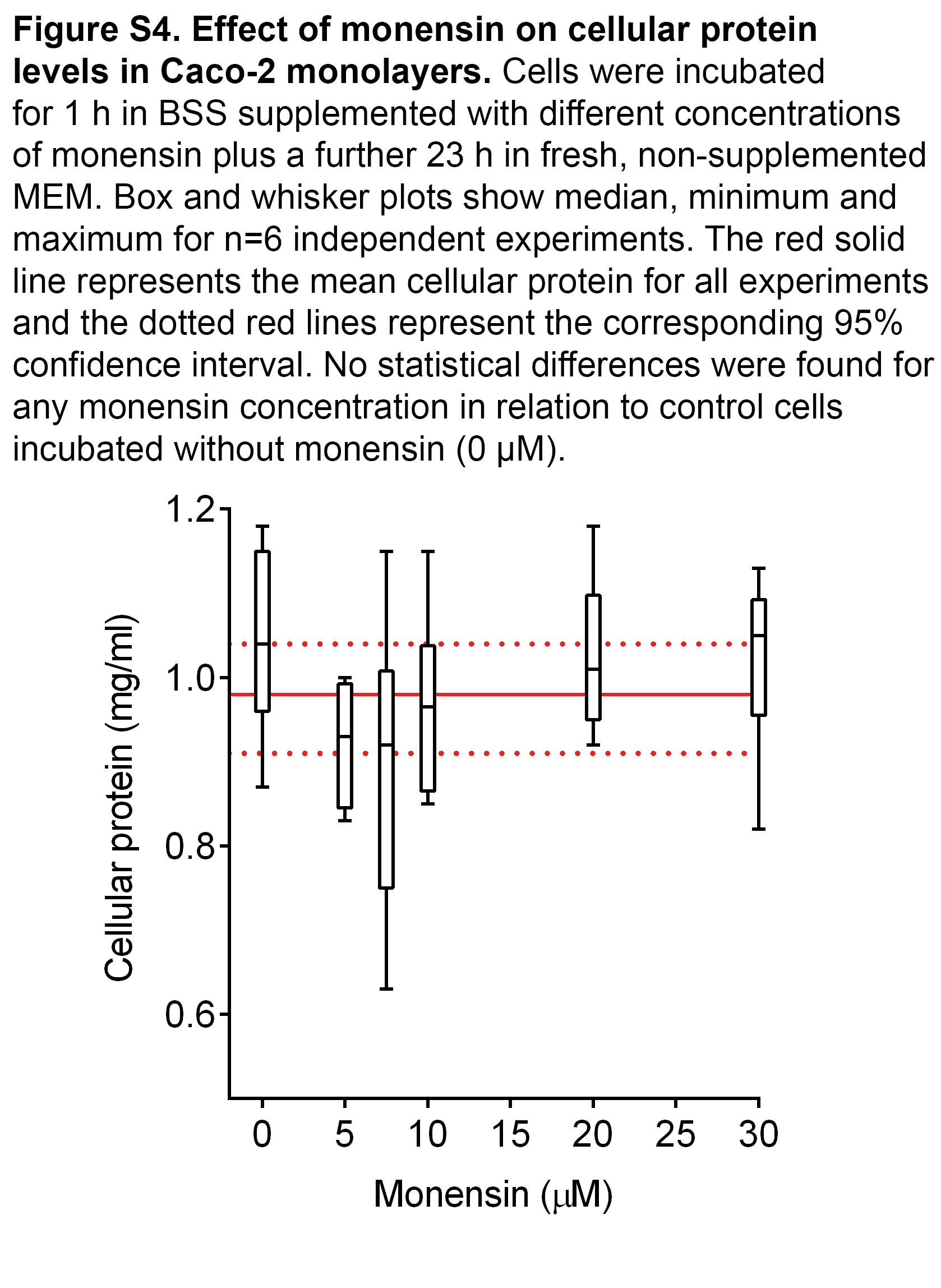

Supplement: Figure S4 — Effect of monensin on cellular protein levels in Caco-2 monolayers. Cells were incubated for 1 h in BSS supplemented with different concentrations of monensin plus a further 23 h in fresh, non-supplemented MEM. Box and whisker plots show median, minimum and maximum for n=6 independent experiments. The red solid line represents the mean cellular protein for all experiments and the dotted red lines represent the corresponding 95% confidence interval. No statistical differences were found for any monensin concentration in relation to control cells incubated without monensin (0 µM). (TIF) [file pone.0081250.s006.tif]

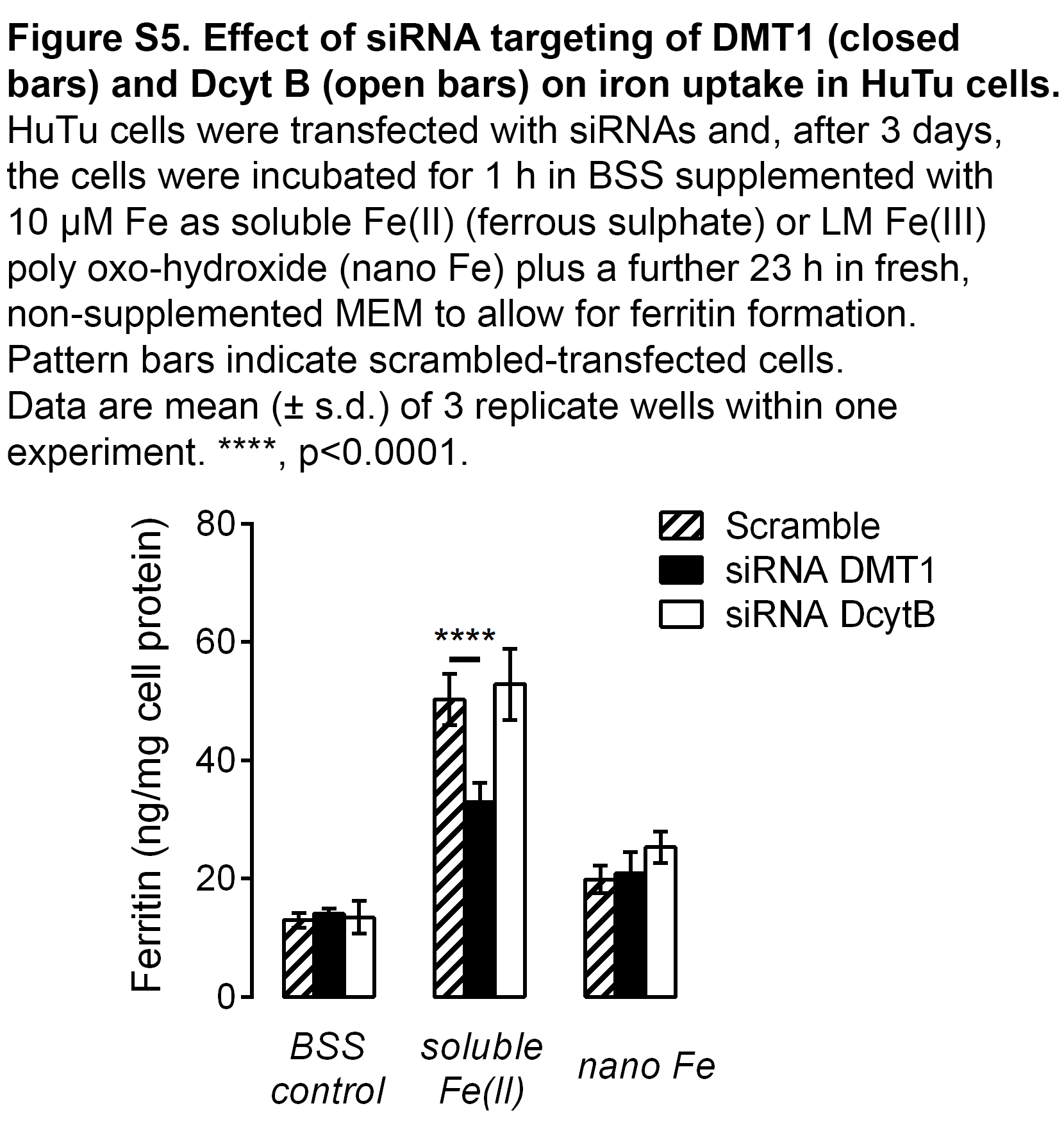

Supplement: Figure S5 — Effect of siRNA targeting of DMT1 (closed bars) and Dcyt B (open bars) on iron uptake in HuTu cells. HuTu cells were transfected with siRNAs and, after 3 days, the cells were incubated for 1 h in BSS supplemented with 10 µM Fe as soluble Fe(II) (ferrous sulphate) or LM Fe(III) poly oxo-hydroxide (nano Fe) plus a further 23 h in fresh, non-supplemented MEM to allow for ferritin formation. Pattern bars indicate scrambled-transfected cells. Data are mean (± s.d.) of 3 replicate wells within one experiment. ****, p<0.0001. (TIF) [file pone.0081250.s007.tif]
